# NF-κB signaling and vesicle transport are correlated with the reactivation of the memory trace of morphine dependence

**DOI:** 10.1186/1746-1596-9-142

**Published:** 2014-07-10

**Authors:** Junyi Ye, Zhaoyang Yang, Candong Li, Meimei Cai, Daizhan Zhou, Qin Zhang, Yiming Wei, Ting Wang, Yun Liu

**Affiliations:** 1 Institute of Biomedical Sciences, Fudan University, Shanghai, PR China; 2 Fujian University of Traditional Chinese Medicine, Fuzhou, PR China; 3 Bio-X Institutes, Key Laboratory for the Genetics of Developmental and Neuropsychiatric Disorders (Ministry of Education), Shanghai Jiao Tong University, Shanghai 200030, PR China; 4 Fujian Medical University, Fuzhou, PR China; 5 Institute for Nutritional Sciences, Shanghai Institutes for Biological Sciences, Chinese Academy of Sciences, Graduate School of the Chinese Academy of Sciences, Shanghai, PR China; 6 Key Laboratory of Molecular Medicine, Ministry of Education, Department of Biochemistry and Molecular Biology, Fudan University Shanghai Medical College, 303 Mingdao Building, 138 Yixueyuan Road, Shanghai 200032, PR China

**Keywords:** CPP, Morphine, Transcriptome, Methylome, NF-κB

## Abstract

**Abstract:**

**Background**

Morphine has been widely used as a clinical anesthetic and analgesic. However, abuse of morphine might result in psychological and physiological dependence. Previous studies have indicated that memory mechanisms play critical roles in morphine dependence.

**Methods**

Morphine dependence was established in mice utilizing place preference conditioning (CPP). We observed changes in the methylome and transcriptome of the nucleus accumbens during the reactivation of the memory trace. We also monitored for changes in the methylome and transcriptome of mice that were acutely exposed to morphine.

**Results**

We detected 165 and 18 differentially expressed genes (DEGs) and 6 and 24 significant methyl-sensitive cut counting (MSCC) windows in the acute morphine treatment and the CPP model, respectively. The changes in the methylome and transcriptome during the acute treatment were mainly caused by a response to the morphine stimulus; most of the DEGs were correlated with hormone or transcription factor activity regulation. The expression levels of *Lcn2* and *Hspb1*, which participate in the activation of NF-κB, were significantly decreased in the CPP morphine treatment model. Besides, the alternative splicing of the curtailed isoform of *Caps1* was significantly increased in the CPP morphine-treated group, and the methylation levels of *Arf4*, *Vapa*, *and Gga3* were decreased. These genes play critical roles in the regulation of the Golgi network.

**Conclusions**

The current study indicates that NF-κB signaling and vesicular transport are correlated with the reactivation of the memory trace in morphine-dependent mice. The results obtained in our study agree with previous observations and identify additional candidate genes for further research.

**Virtual Slides:**

The virtual slide(s) for this article can be found here: http://www.diagnosticpathology.diagnomx.eu/vs/1196707364133126

## Background

Opioids are widely used as clinical anesthetics and have been irreplaceable for the treatment of severe pain for decades. Chronic illicit use or overdose in clinical settings may result in side effects including development of tolerance and withdrawal [1]. The sudden cessation of morphine use produces an intense withdrawal syndrome in both humans and animal models [1]. Opioid addiction is characterized by compulsive drug-taking behavior and high rates of relapse. The addiction not only causes medical, social and economic problems but also severely harms the health of drug abusers [2-4].

Inhuman addicts, the correlation of drug use with environmental cues significantly increases the risk of relapse [5]. Previous studies indicate that memory mechanisms play critical roles in addiction [6,7]. The reactivation of opioid drug produces an incentive motivational state that promotes drug craving and in turn promotes seeking environmental stimuli associated with receipt of opioid drug [8,9]. Environmental stimulus may change DNA methylation, which was regarded as highly stable epigenetic mark to ensure transcriptional gene silencing. However, Guo et al. and his colleague discovered that the methylome was not as stable as general belief in terminally cells, only one electroconvulsive stimulus could rapidly modify a large number of CpGs by neuronal activity in brain [10]. Therefore, We suggest that a rapid modification of CpGs may occur during the reactivation of the memory trace in morphine-dependent mice. The primary aim of the present study was to characterize methylome and transcriptome changes in nucleus accumbens to study the potential mechanism of morphine dependence and drug seeking after environmental stimulus. In addition, consideringmorphine is widely utilized as clinical anesthetic, we are curious about whether once morphine injection will change the CpGs methylation as electroconvulsive stimulus [10]. Hence, we also determined the changes in methylome and transcriptome after acute morphine injection. The mice in CPP experiment and acute morphine treatment were in different morphine states: during the acute experiment the mice were on-morphine, while during the conditioned place preference experiment they were off-morphine. In present study, we also compared the transcriptome and methylome differences between the two states.

## Methods

### Animals

KM mice (Fujian University of Traditional Chinese Medicine, China) were used in the experiments. Twelve 8-week-old male mice were used for the acute morphine treatment, whereas nine male mice were used in conditioned place preference (CPP) experiments. The mice were kept under standard conditions on a 12 h light–dark cycle and had free access to food and water. Ethical approval was obtained from the Shanghai Fudan University Ethical Committee (Shanghai, China).

### Drugs

Morphine hydrochloride was obtained from Shenyang No.1 Pharmaceutical (Shenyang, China) and dissolved in physiological saline (10 mg · mL^-1^).

### Acute morphine treatment

The mice were randomly divided into two groups (morphine and saline). To remove interference caused by the abdominal injections, all the animals received injections of saline for 3 days. On day 4, the animals in the morphine group were injected with 10 mg · kg^-1^ morphine (about 300 μl), whereas the control animals received an equal volume of saline. The animals were euthanized by decapitation after 4 hours. The nucleus accumbens was dissected immediately, rapidly frozen in liquid nitrogen, and stored at -80°C until analysis.

### Conditioned place preference

The rewarding effects of morphine were examined using CPP methods [11]. The testing apparatus consisted of two compartments. The compartments were identical in size (15 cm × 25 cm × 30 cm). A passageway connected the compartments. One compartment was randomly assigned to the drug treatment, and the other one was paired with the saline treatment. The mice were randomly divided into two groups (six for morphine and three for saline). The CPP protocol consisted of a 10-day schedule with three distinct phases: preconditioning, conditioning, and postconditioning. Because of the study objective, a final stage, the reactivation of the memory trace, was included on day 11, 1 day after the place preference conditioning.

### Preconditioning phase

During this phase (day 1), each animal was placed in the middle passageway of the apparatus, and the guillotine door was raised to allow access to the entire apparatus for 15 min. The residence time of the mouse in each compartment was accurately measured by head position using JL-Behv-CPP version 2.0 software (Shanghai Jiliang Software Technology), an automated video-tracking system used for behavioral experiments. Most animals spent approximately the same amount of time in each compartment. No animals showed a strong unconditioned preference (>540 s).

### Conditioning phase

This phase (from day 2 to day 9) consisted of eight 45-min sessions. The mice in the morphine group received an abdominal injection of 25 mg · kg^-1^ morphine on days 2, 4, 6, and 8 and the same volume of saline on days 3, 5, 7, and 9. The control mice received saline every day. Immediately after the saline or morphine injections, each animal was confined in its assigned compartment for 45 min by closing the guillotine door.

### Postconditioning phase

In this phase (day 10), the animal preferences were tested. No injection was given. For testing, the animals were placed in the middle passageway of the apparatus with the guillotine door raised and were allowed free access to both compartments for 15 min. The residence time of the mice in each compartment was accurately recorded, as in the preconditioning phase. To compute the ratio of the time that the animals spent in the drug-paired side to the total time spent in the apparatus, a CPP score was used [12].

### Reactivation of the memory trace

The animals were placed in the drug-paired compartment by closing the guillotine door for 10 min. The animals were euthanized 4 hours after the reactivation of the memory trace, and the nucleus accumbens was excised immediately, rapidly frozen in liquid nitrogen, and stored at -80°C until analysis.

### Library preparation

Total RNA was extracted with TRIzol reagent according to the manufacturer’s instructions (Invitrogen). For mRNA-sequence (mRNA-seq) sample preparation, the Illumina standard kit was used according to the TruSeq RNA Sample Preparation Guide v2 (Illumina, Supplementary).

### Sequencing and primary analysis

Sequencing was performed on an Illumina HiSeq2000 sequencing system. The raw RNA-seq data were filtered with Trimmomatic version 0.20 [13] according to the following criteria: 1) reads containing sequencing adaptors were removed, 2) nucleotides with a quality score lower than 20 were removed, and 3) reads shorter than 36 were discarded.

### Mapping of RNA-seq reads

The clean reads were then aligned to the University of California at Santa Cruz (UCSC)mm9 reference genome using TopHat v2.0.3 software [14], which uses Bowtie v0.12.8 to perform the alignment [15]. The prebuilt index of the UCSC mm9 genome was downloaded from the TopHat home page (http://tophat.cbcb.umd.edu/index.html) and used as the reference genome. First, TopHat splits the reads into shorter segments. Next, Bowtie reports successfully mapped segments that have no more than 2 mismatches. TopHat allows for multiple alignments per read (up to 20 by default) when mapping the reads to the reference. TopHat builds a database of potential splice junctions and confirms these junctions by comparing the previously unmapped reads with the database of putative junctions. The default parameters for the TopHat program were used.

### Differentially expressed gene testing

The alignment .SAM files produced by TopHat served as inputs for the Cuffdiff v2.0.2 program [16], along with the downloaded UCSC gene transfer format (GTF) file. Cuffdiff re-estimates the abundance of the transcripts listed in the GTF file using alignments from the SAM file and concurrently tests for differential expression. In Cuffdiff, only the genes with a “q_value” less than 0.05 were marked as “OK,” which indicated differential expression. The DEGs, as determined by this analysis, were visualized using CummeRbund (http://compbio.mit.edu/cummeRbund/).

### Detection of differential exon-skipping events using mixture-of-isoforms analysis

It is well known that alternative splicing (AS) of a gene locus can cause expression of multiple isoforms. Whole genome screening to identify AS events has the advantage of the RNA-seq methods, generally unavailable with microarray technologies.

In this study, a mixture-of-isoforms (MISO) analysis [17], which employed Bayesian inference to compute the probability that a read originated from a particular isoform, was utilized to detect exon-skipping events. The read alignment files .BAM produced by TopHat, as described above, and the prebuilt mouse genome (mm9) alternative event annotations downloaded from the MISO manual page were utilized as input. We performed the MISO analysis according to the procedure outlined in the MISO manual, except that the Bayes factor, which corresponds to the odds of differential expression, was set to 1,000 to more strictly filter differentially expressed events. The percentage of mRNAs that were spliced to include exons, called the Psi” value (Ψ), was calculated. See Katz et al. [17] for a description of these values and their method of computation. The ΔΨ value was the difference between the Ψ values of the two groups in each comparison.

### Differentially expressed gene and exon-skipping event validation

Differential gene expression was validated by real-time quantitative polymerase chain reaction (qRT-PCR) methodology using the ViiA 7 Real-Time PCR System (Applied Biosystems). Reverse transcription was performed with Superscript II reverse transcriptase according to the manufacturer’s protocol (Invitrogen). We selected 4 DEGs from the CPP model for DEG validation. We also designed primers that contain the junction to validate the exon-skipping events of *Caps1*. The PCR reactions included 1x SYBR Green Taqman Gene Expression master mix (Applied Biosystems), 100 nM · μl^-1^ forward and reverse primer, and 5 μl of 30-fold diluted cDNA template. The cycling conditions for all the primer pairs were 95°C incubation for 10 min, followed by 40 cycles of 95°C for 15 sec, and 60°C for 1 min. The primer sequences are listed in Additional file 1: Table S1. For quality control, all the reactions were performed in duplicate, and the results were checked for agreement to capture intra-assay variability.

The expression levels of each target gene were compared between the drug and saline groups at all time points. The data were normalized using *Gapdh*, and the relative gene expression was calculated using the 2^-(ddCt)^ method and assessed by Student’s *t*-test. All the tests were performed using R version 2.14.0 with P < 0.05 required for statistical significance.

### Functional analysis of differentially expressed genes

The differentially expressed genes (DEGs) identified by Cuffdiff for the acute model were used as inputs for the analysis of gene ontology enrichment using DAVID [18,19]. However, redundancy is particularly problematic with enrichment results obtained in this manner because the GO terms are derived from hierarchical functional annotation systems. Children terms that are partially redundant with their parents are quite prominent. The present study used EnrichmentMap, an open-source plug-in for the Cytoscape network visualization and analysis software, to integrate the enriched GO terms as a weighted similarity network [20,21].

### Genomic DNA extraction

For each sample, genomic DNA extracted from six mice was pooled. The DNA was extracted using the QIAamp DNA Mini Kit (Qiagen). The DNA length was determined by an Agilent 2100 bioanalyzer to ensure integrity. The genomic DNA was then fragmented using a Gene Machines Hydroshear apparatus (Harvard Apparatus) at set point12 for 40 cycles.

### Methyl-sensitive cut-counting library construction

The methyl-sensitive cut-counting (MSCC) libraries were constructed following the method of Guo et al. [10], with a few changes. See the Additional file for details.

### Sequencing and data analysis for the methyl-sensitive cut-counting assay

First, the MSCC libraries were pooled. Sequencing was performed on an Illumina HiSeq2000 sequencing system. The sequencing reads contain an 18-bp “tag” and a part of the sequence of adaptor A. The index sequences within adaptor A were used to distinguish different MSCC libraries. After adaptor removal, the 18-bp tags were mapped to the dataset of all possible 18-bp tags in the mouse genome (mm9) for each library using MOM software [22]. The reads that mapped uniquely to the dataset and that contained fewer than 2 mismatches were accepted. For each CCGG site, the sequencing reads from the *Hpa*II library came from CCGG sequences, and the reads from the inverse library came from CmCGG or ChmCGG sequences. After normalizing for the number of reads in both libraries using the counts of standard DNA, the methylation level of each site was calculated. The sequenced sites were then mapped to known genes and CGIs using relevant tables downloaded from the UCSC Genome Browser (http://genome.ucsc.edu/).

To avoid false positives because of adjacent CG sites with similar DNA methylation levels, we first scanned the genome using 200-bp consecutive windows with 50-bp overlaps and known CGIs as units to find differentially methylated regions. For each of these units (>3 sequenced CpGs), the average methylation level of the mapped CpGs was calculated. A P value was also assigned using Student’s paired *t*-tests to compare each unit between the different samples.

### *Hpa*II-qPCR validation

For the validation of the *Hpa*II-based methylation-sensitive qPCRs, 400 ng of genomic DNA was mock treated or digested with *Hpa*II for at least 4 h. After heat inactivation at 65°C for 20 min, the same volumes of products were used as templates for the qPCRs with the primers (Additional file 1: Table S1). The methylation fraction was calculated as 2^(Ct_mock_-Ct_HpaII_).

## Results

### Conditioned place preference

The results showed that the morphine-treated animals spent significantly more time in the drug-paired side during the postconditioning than the preconditioning phase (assessed by student’s t-test, P < 0.05, Table 1), whereas the control animals showed no significant preference (assessed by student’s t-test, P > 0.05, Table 1).

**Table 1:** Ratio of the time that the animals spent in the drug-paired during post-conditioning and pre-conditioning phase

	Radio of the time*	
**Group**	**Pre-conditioning**	**Post-conditioning**
Morphine group	0.2860 ± 0.1598	0.8560 ± 0.1297
Saline group	0.2000 ± 0.2095	0.1967 ± 0.1950

*Ratio of time means the animals spent in the drug-paired side to the total time spent in the apparatus.

### Global properties of the expression profile

We utilized RSeQC [23] for quality control in present study (Additional file 1: Figure S1). The normalized expression level of each gene was measured as fragments per kilobase per million (FPKM) mapped reads. In our study, we detected more than 15,000 expressed genes in each sample. We further compared the expression profiles between the saline and the morphine groups. The global profiles of gene expression were highly correlated; both of the Pearson correlation coefficients exceeded 0.98 (Additional file 1: Figure S2a). With an FDR < 0.05 and a fold change > 1, we detected 165 and 18 DEGs in the acute morphine treatment and the CPP models, respectively (Additional file 1: Figure S2b, Additional file 1: Table S2 and Additional file 1: Table S3). Among these DEGs, *Lcn2* and *Hspb1* participated in the regulation of NF-κB pathway activation, which plays a complex role in morphine dependence. Especially, recently study discovered that LCN2 could trigger the neuronal NF-κB response [24] and participate in dendritic spine shape changes associated with psychological stress [25].

### Analysis of alternative splicing events

#### Acute morphine treatment

Using the MISO analysis, we detected 10,608 and 10,692 exon-skipping events in the saline and morphine groups, respectively. We next compared the differential exon-skipping events (DESs) between the saline and the morphine groups. We filtered with the recommended parameters except that the Bayes factor was set at 1,000, a rather conservative value, and found 6 DESs that located in 3 genes (Table 2).

**Table 2:** The DES between saline and morphine injection

	**Events**	**Gene**	**Bayes factor**	**ΔΨ**
Acute morphine treatment	chr6:93647188:93647481:-@chr6:93644002:93644205:-@chr6:93636776:93636869:-	*Magi1*	6.39E + 07	-0.38
	chr6:93647188:93647481:-@chr6:93644002:93644202:-@chr6:93636776:93636869:-	*Magi1*	1.71E + 05	-0.39
	chr6:93647188:93647481:-@chr6:93644002:93644169:-@chr6:93636776:93636869:-	*Magi1*	6.38E + 04	-0.4
	chr19:7542301:7542357:-@chr19:7530790:7532913:-@chr19:7509421:7509628:-	*Rtn3*	5.60E + 04	-0.44
	chr19:7542301:7542357:-@chr19:7530790:7531950:-@chr19:7509421:7509628:-	*Rtn3*	1.31E + 04	-0.39
	chr1:132595256:132595320:-@chr1:132593594:132594662:-@chr1:132585620:132587725:-	*Pfkfb2*	5.05E + 03	-0.37
CPP	chr2:164612198:164612355:+@chr2:164617058:164617068:+@chr2:164617213:164618270:+	*Acot8*	1.00E + 12	-0.72
	chr3:87965113:87965303:+@chr3:87965703:87965723:+@chr3:87966880:87967009:+	*Mef2d*	1.00E + 12	0.39
	chr10:127917907:127918060:+@chr10:127918738:127918830:+@chr10:127919139:127919258:+	*Smarcc2*	4.84E + 06	0.22
	chr14:13319039:13319107:-@chr14:13318155:13318172:-@chr14:13305923:13306029:-	*Caps1*	2.29E + 04	0.51

ΔΨ was the difference between the Ψ value, which stood for percent spliced for the exon inclusion, of the two groups in each comparison.

### CPP model

**Figure 1:**
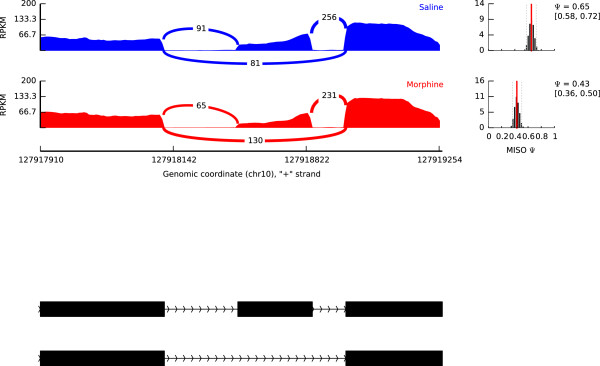
**RNA-seq reads mapped to the UCSC mm9 of *Caps1*.** The results are shown in blue for saline and in red for morphine. The counts of reads spanning exon junctions are shown. The “Psi” values (Ψ), which represent the percentage of mRNAs spliced to include exons, were calculated.

We detected 9162 and 9368 exon-skipping events in the saline and the morphine groups, respectively, and compared the DESs of these groups. When the same conditions as those of the acute morphine model were used, we found 4 DESs (Table 2). *Caps1*, which included one of these DESs (Figure 1), encodes a membrane protein that serves to modulate neuropeptide-containing dense-core vesicles (DSVs) at secretion sites, such as nerve terminals [26]. DSVs can store opioid peptides, such as dynorphin and enkephalin. Opioid peptides such asdynorphin can decrease dopamine release by binding to κ-opioid receptors (KORs) on dopamine nerve terminals, which leads to drug tolerance and withdrawal symptoms [27].

### Validation of DEGs and DESs

**Figure 2:**
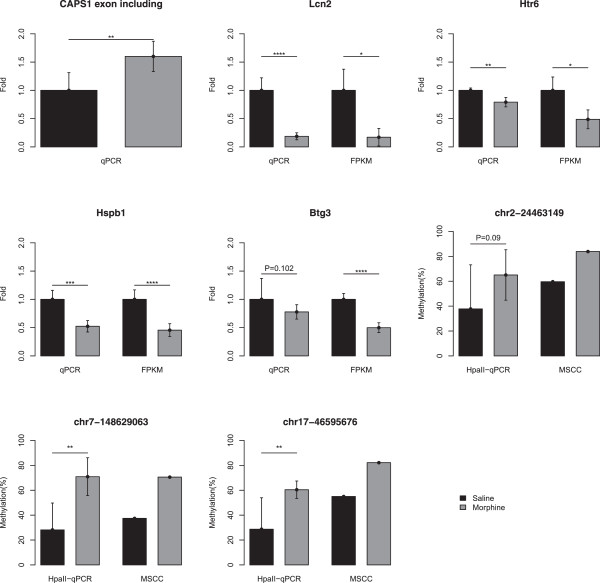
**The DEGs detected by RNA-seq were confirmed by qRT-PCR.** The MSCC sites were confirmed by *Hpa*II-qPCR. The expression level or methylation level of each gene was normalized to the level of saline. * Denotes P < 0.05, **denotes P < 0.01, *** denotes P < 0.001, and **** denotes P < 0.0001.

To experimentally confirm the DEGs and DESs identified by RNA-seq, the expression levels of the identified genes were validated in each sample by quantitative real-time PCR (qRT-PCR). We randomly selected 4 DEGs from the CPP model for validation, namely, *Hspb1*, *Htr6*, *Lcn2*, and *Btgs*. We used *Gapdh* as the endogenous reference. The qRT-PCR results showed nearly identical changes in gene expression to those detected via the RNA-seq technique, as shown in Figure 2.

We also designed a pair of primers that contain the exon junction to validate the exon-skipping event of *Caps1*. The result showed that the exon inclusion isoform was increased in the morphine group, which agrees with the results of the MISO analysis (Figure 2).

### Global properties of DNA methylation

In present study, we detected more than 1,000,000 CCGG sites in each sample. The data was summarized in Additional file 1: Table S4. To ensure accuracy and CG coverage in all the samples, we chose CCGG sites with more than 40 sequencing reads to do the following analysis. The selected sites were mapped to the known genes of the UCSC mm9 mouse genome. The majority of the sites are located in intron and intergenic regions (Additional file 1: Figure S3a). Additionally, due to the randomness of NGS, the distribution of the CCGG sites with over 40 reads was very similar to that of all the CCGG sites in the mouse genome. Further, according to the data of Guo et al. [10], the distribution of CCGG sites corresponds to all CG sites; therefore, the methylation of sites with over 40 reads may satisfactorily reflect the genome-wide methylation state.

The calculated methylation level of the CCGG sites was plotted based on the relative positions of the nearby genes (Additional file 1: Figure S3b). The moving average is also shown. The regions near transcription start sites (TTSs) showed significant hypomethylation. We also mapped the CCGG sites with more than 40 reads to known CGIs and CGI shores. Approximately half of the sites were located in CGIs or CGI shores (Additional file 1: Figure S4a). As expected, the CCGG sites within the CGIs were poorly methylated, whereas those sites located outside of CGIs were hypermethylated (Additional file 1: Figure S4b). Additionally, the CGI shores showed an intermediate methylation level.

### The methylation pattern is closely related with gene expression

**Figure 3:**
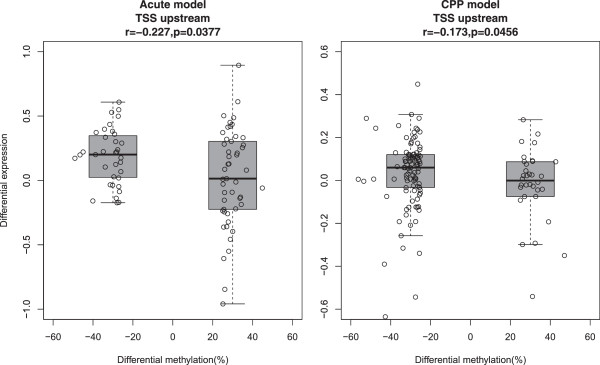
**The DNA methylation level upstream of TSSs is closely related to gene expression.** Correlation between methylation changes of CpGs and mRNA level changes of associated genes between morphine group and control (P values, Pearson's correlation test). Each dot stood for a CpG site with a cutoff of 25% methylation change.

It is well known that hypermethylation in a promoter region inhibits gene expression. Therefore, we next determined whether changes in the CpG sites upstream of TSSs were correlated with changes in gene expression (Figure 3). Increases in methylation in regions upstream of the TSSs were correlated slightly but significantly to decreases in gene expression in both models.

### Analysis of the methylation changes within 200-bp windows

#### ***Acute morphine treatment***

Using a cutoff of 25% and a P value <0.05 (Additional file 1: Figure S5), we detected 6 windows with significantly different methylated regions in the morphine compared with the saline group. These significant windows located within 6 genes. Among these gene, *Lrdd* and *Nfkbib* were correlated with NF-κB signaling regulation [23,28].

### CPP model

Using the same method as that used for the acute morphine treatment, we detected 24 windows that were located within 22 genes and differed significantly between the morphine and the saline groups. Among these genes, *Vapa*, *Gga3*, and *Arf4* are correlated with vesicle trafficking. GGA proteins are recruited to membranes via their direct interaction with GTP-bound ARFs. ARFs play pivotal roles in membrane traffic, cell signaling, and act in cytoskeletal rearrangements. In mammalian cells, there are six ARF families. ARF4 belongs to class II ARFs and reportedly regulates protein and membrane transport in the Golgi complex [29].

### *Hpa*II-qPCR analysis

To experimentally confirm the significant sites of MSCC, three sites located in different genes were validated in each sample by *Hpa*II-qPCR. The *Hpa*II-qPCR results showed nearly identical changes in methylation to those detected via MSCC (Figure 2).

## Discussion

The present study assessed changes in the transcriptome and methylome when the memory trace of morphine-addicted mice was reactivated. In the CPP experiments, the mice that were injected with morphine showed a significant place preference. The expression levels of 18 genes were significantly changed. More than half of these genes are correlated with G protein-coupled receptor, neurotransmitter, dendritic spine formation, and NF-κB signaling. A previous study discovered that lipocalin-2 (LCN2), an iron-related protein, participates in determining spine morphology and regulates neuronal excitability. In addition, LCN2-null mice displayed anxious and depressive-like behaviors as well as cognitive impairment in spatial learning tasks [30], in accordance with the symptoms of morphine addiction. LCN2 also triggers the neuronal NF-κB response [24] and has recently been shown to exist in neurons and participate in dendritic spine shape changes associated with psychological stress [25].

In the present study, the expression levels of *Lcn2* and *Hspb1*, which both produce protein products known to enhance NF-κB pathway activation, decreased after the CPP experiment. NF-κB signaling plays a complex role in morphine dependence. However, previous studies indicated that the inhibition of NF-κB disrupted morphine-related memory reconsolidation and blocked place preference conditioning [31,32]. Thus, these previous studies appear to contradict our data. Nevertheless, morphine can also inhibit the activity of NF-κB. In fact, the stimulation of neuronal cells with morphine leads to a transient activation of NF-κB and a strong induction of c-Fos [33,34], one of the components of activator protein-1 (AP-1) [35]. AP-1 and NF-κB itself result in the opioid-induced inhibition of NF-κB. Thus, such a transient activation of NF-κB may play a critical role in the rewarding effects of morphine.

With the MISO analysis, we detected 4 exon-skipping events in the CPP model. Among them, *Caps1* encodes an evolutionarily conserved calcium-binding protein that follows ATP-dependent priming and is essential for exocytosis of neurotransmitters from synaptic terminals [36], especially for large dense-core vesicles (LDCVs) [37,38]. According to the annotation for *Caps1* in the UCSC and RefSeq databases, only one isoform contains the DES that had been defined in our study. However, this isoform is truncated onthe 3’ and 5’ termini. To the best of our knowledge, no previous study has reported whether this isoform functions similarly to the other integrated isoforms, but we speculated that such a shortened isoform might be dysfunctional for ATP-dependent priming or membrane localization.

Table 2 shows that the ΔΨ values of *Caps1* increased, indicating that the expression level of the deficient isoform increased and the exocytosis of neurotransmitters may have been depressed. Neurotransmitters are packaged into two classes of secretory vesicles: small clear synaptic vesicles and LDCVs. The LDCVs contain fast-acting neurotransmitters, such as glutamate, and slower acting peptides, such as opioid peptides, or amines, such as dopamine [39]. Glutamate is a well-known NF-κB signaling activator [24,40,41], which is important for synaptic plasticity and has been described above. Endogenous opioids regulate emotion, pain sensitivity and sexual activity. The dysregulation of endogenous opioid systems, including dopamine systems, leads to changes in the physiological state to achieve a new hedonic set point [42,43].

The MSCC analysis for the CPP experiment discovered 24 significantly different windows between the saline and the morphine groups. Among the affected genes, *Arf4*, *Arl4c*, *Gga3*, and *Vapa* are correlated with intracellular transporters. *Arf4* is a member of the ARF gene family whose members have been determined to be important regulators in vesicle trafficking. *CAPS1* interacts especially with *ARF4/ARF5* and further regulates dense-core vesicle (DCV) trafficking in the trans-Golgi network. However, knockdown of either *Caps1* or *Arf4* expression reduced the secretion of DCVs [44]. Considering the possible inactivation of *Caps1* suggested by the results of the MISO analysis, we are not sure that the change in methylation of *Arf4* could regulate DCV secretion. ARF4 recruits GGA and adaptor proteins along with coatomer (COPI) to the Golgi membrane to regulate intracellular transport [45,46]. The GGA proteins regulate the trafficking of proteins between the trans-Golgi network and the lysosome [47]. COPI is a protein complex essential to the retrograde transport of proteins from the *cis*-Golgi back to ER [48,49]. Previous study reported that the dysfunction of vesicle transport might lead to disease, such as pediatric gliomas [50]. Nevertheless, we have found no articles that directly correlate intracellular transport and morphine dependence, which may be influenced by neurotransmitter release in the synapse. This unexplored area may provide a productive direction for the study of morphine dependence.

**Figure 4:**
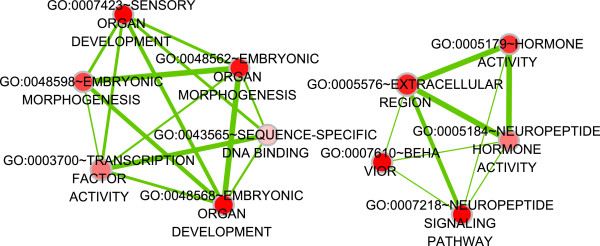
**Network of enriched GO terms derived from the DEGs of the acute model.** The enriched GO terms are organized as a weighted similarly network, with nodes representing enriched GO terms and internode links representing the overlap score calculated from the number of genes shared by two GO terms.

We also determined the changes in the expression profile and in the alternative splicing of mRNA after acute morphine injection. Because of the large number of DEGs in the acute treatment, we examined these DEGs with DAVID, a functional enrichment analysis program, and obtained 11 statistically significantly enriched GO terms. Using EnrichmentMap, the GO terms were integrated as a weighted similarity network (Figure 4). Most DEGs from the acute treatment group are correlated with organ development, signaling, and neuropeptides. Nevertheless, the GO analysis was considered to provide an indication of the functional association between gene groups rather than a demonstration of direct correlation with the process described by the GO terms. Our data identified several genes of functional importance to the prolonged effects of morphine, such as *Avp*. The Avp/V1b receptor system is a critical component of a pathway responsible for the influence of negative emotional states on drug-seeking behavior [51].

In the acute model, we used the MISO analysis to detect 6 DESs that are located in 3 genes. Among these genes, *Magi1* encodes a member of the membrane-associated guanylate kinase homologue (MAGUK) family. MAGI-1 is a tight junction-associated multidomain protein, which bears six PDZ domains [52]. The tight junction may act as a platform for trafficking and signaling protein complexes [53]. Two splice variants of MAGI-1 localize to cell-cell junctions, indicating that MAGI-1 may play a critical role as a scaffolding protein at cell-cell junctions. The annotation in the RefSeq database indicates that there are four *Magi1* isoforms in all: three of them entail the inclusion of three different exon lengths, and one entails exon exclusion. Table 2 shows that the ΔΨ values for three events of *Magi1* decreased, which indicates that the expression level of the exon exclusion isoform, namely NM_010367, increases after acute morphine treatment. The effects of this exon exclusion isoform require further investigation.

The MSCC analysis identified 6 windows with significant changes in methylation following the acute morphine treatment. Within these identified windows, *Lrdd* encodes a protein that contains a leucine-rich repeat and a death domain (DD). The loss of *Lrdd* limits NF-κB activation [54]. In addition, *Nfkbib* showed a significant change in methylation. The protein encoded by *Nfkbib* participates in the regulation of NF-κB activation through the ubiquitination pathway [28]. NF-κB signaling plays a complex role in morphine dependence, as discussed above.

In summary, the present study identifies methylome and transcriptome changes occurring during memory trace reactivation in morphine-dependent mice and compares these changes to those occurring in mice treated acutely with morphine. The results of the two morphine treatment models were entirely different due to different morphine states. The changes in the methylome and transcriptome of the mice treated acutely were mainly caused by a response to the morphine stimulus. Especially for the transcriptome, most of the DEGs were associated with hormone regulation or transcription factor activity. However, most of the DEGs and the significant MSCC windows in the morphine-dependent mice (the CPP model) indicated that the regulation of NF-κB signaling and vesicular transport is involved in the reactivation of the memory trace of morphine dependence.

In addition to NF-κB signaling and vesicular transport, because of the complex mechanism of morphine dependence, acetylcholine, dopamine systems, GABA signaling, and other pathways participate in this dependence. For example, in the CPP model, we observed a significant increase in the expression of the nicotine acetylcholine receptor in the present study and also discovered many DEGs and significant MSCC windows that are correlated with ubiquitination. Even Gga3, which participates in the regulation of intracellular transport, has two ubiquitin-binding motifs [55]. We cannot discuss all the possible mechanisms of morphine dependence in this article, but the results obtained in the present study expand our knowledge of these mechanisms and identify a more diverse group of potentially involved genes for further research.

## Conclusions

The current study indicates that NF-κB signaling and vesicular transport are correlated with the reactivation of the memory trace in morphine-dependent mice. The results obtained in our study agree with previous observations and identify additional candidate genes for further research.

## Abbreviations

CPP: Place preference conditioning; DEG: Differentially expressed genes; MSCC: Methyl-sensitive cut counting; FPKM: Fragments per kilobase per million; DES: Exon-skipping events; AS: Alternative splicing; DCV: Dense-core vesicle.

## Competing interests

The authors declare no competing financial interests.

## Authors’ contributions

Conceived and designed the experiments: JY ZY YL. Performed the experiments: JY MC TW FL DZ. Analyzed the data: JY MC ZY. Contributed reagents/materials/analysis tools: CL QZ YM. Wrote the paper: JY ZY YL. All authors read and approved the final manuscript.

## Supplementary Material

Additional file 1 This additional file includes transcriptome and MSCC library preparation methods, and supplementary figures and tables. Click here for file
