# Supplementary material for: NF-κB signaling and vesicle transport are correlated with the reactivation of the memory trace of morphine dependence
Source: Diagn Pathol. 2014 Jul 10;9:142. doi: 10.1186/1746-1596-9-142 (PMC4227096; doi:10.1186/1746-1596-9-142)
Supplement: Additional file 1 — This additional file includes transcriptome and MSCC library preparation methods, and supplementary figures and tables. [file 1746-1596-9-142-S1.docx]

**Supplementary**

**Transcriptome library preparation**

Briefly, 4μg of total RNA from each sample was used for polyA mRNA selection using poly T oligo-conjugated magnetic beads by two rounds of purification, followed by thermal mRNA fragmentation. The cleaved mRNA fragments were reversely transcribed and then converted into double strand cDNA. Following end repair and A tailing, adapters complementary to sequencing primers were ligated to the ends of DNA fragments. Finally, the libraries were enriched using 12 cycles of PCR and purified with the Ampure (Beckman) magnetic beads.

**MSCC library construction**

For each of the samples, two libraries were constructed. Two custom adaptors that contain 5’ CG overhang and 3’ NN overhang respectively were created. For HpaII library, 2μg genomic DNA combined with standard DNA was digested with HpaII (NEB) for 2h. Adaptor A was ligated to the resulting fragments. The reaction products were then incubated with Bst DNA polymerase (NEB) for 20 min. After digestion with MmeI (NEB), adaptor B was added to the reaction mixture and incubated with T4 DNA ligase (NEB) for overnight. The products were purified with Agencount AMPure XP Beads (Beckman) and then run on a 2% E-Gel® EX Gel (Invitrogen). The target band at ~140bp was purified with QIAquick Gel Extraction Kit (Qiagen). A 8 cycles PCR was performed on the purification products. For the inverse library, after HpaII digestion in the first step, the fragment ends were deactivated by incubated with Antarctic Phosphatase (NEB). The products were digested with MspI and then treated with the same procedure as HpaII library.

**Supplementary Figure S1**

**Figure S1.** Coverage uniformity over gene body without bias. Abscissa means the relative position of gene.**Supplementary Figure S2**

**a**

**b**

**Figure S2.** Differential expression analysis of morphine treatment mice. (a) The scatter plot for global expression between Saline and morphine group; the Pearson correlation coefficient is shown. (b) Volcano plots for all genes in each comparison. The red dots indicate DEGs with q value less than 0.05.

**Supplementary Figure S3**

**a**


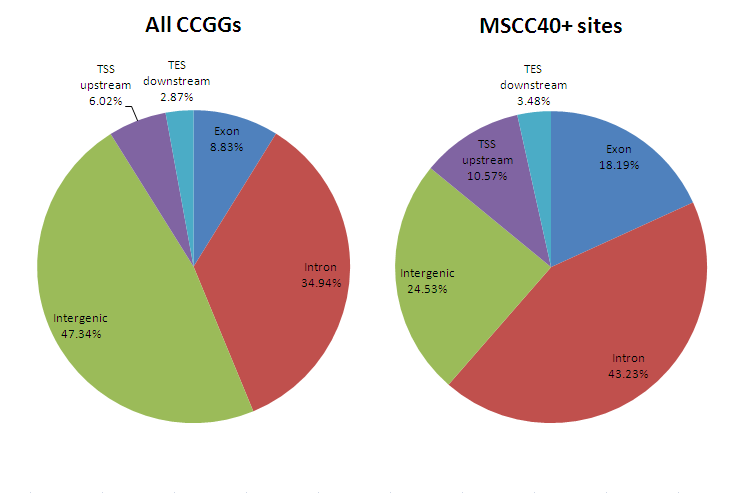


**b**


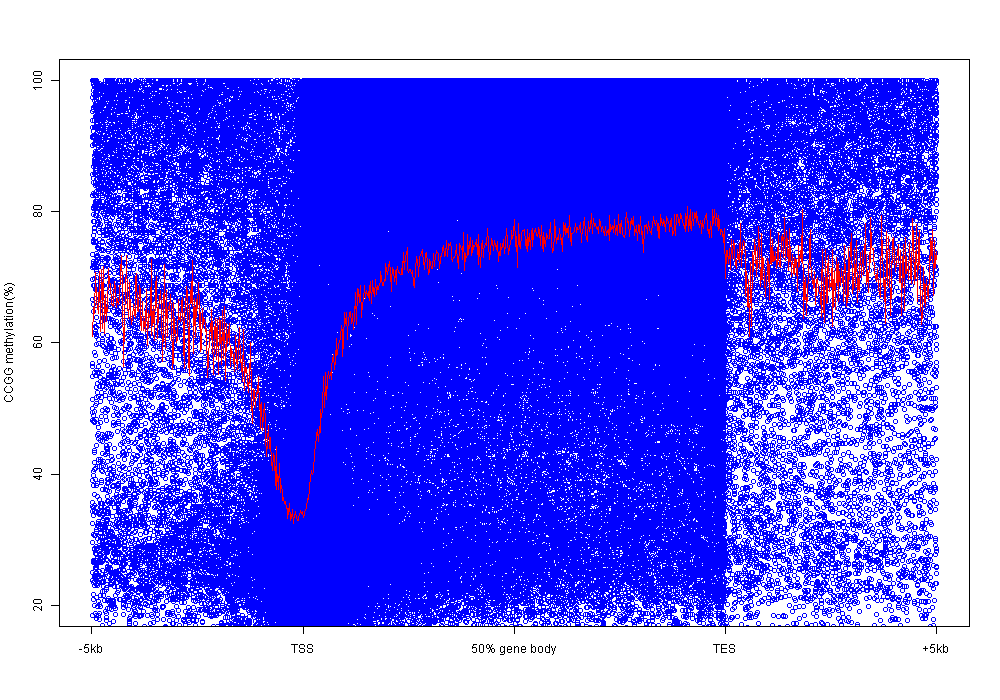


**Figure S3.** Global properties of DNA methylatio. (a) The distribution of all the CCGG sites and those with over 40 reads. CCGG sites with 40+ reads show similar distribution with all CCGG sites. (b) The distribution of CCGG methylation level from the perspective of genes (blue hollow dots). The red line shows the moving average of methylation level.

**Supplementary Figure S4**

**Figure S4.** (a) The CCGG sites with 40+ reads were mapped to CGIs and CGI shores. About half of the CCGG sites are located in CGIs or CGI shores. (b) Overall methylation level of CCGG sites located in the respective regions.**Supplementary Figure S5**

**Figure S5.** Comparison of genome-wide DNA methylation of mouse nucleus accumbens between CPP and saline groups. The cyan dots indicate the average methylation level of the 200-bp windows.

**Table S1. The primers for validation**

|  | **Sequence** |
| --- | --- |
| *Gapdh-F* | 5’-AACTTTGGCATTGTGGAAGG-3’ |
| *Gapdh-R* | 5’-ACACATTGGGGGTAGGAACA-3’ |
| *Btg3-F* | 5’-TCAGGGTCCTCTTCCTCAGA-3’ |
| *Btg3-R* | 5’-GGGCGGAATGGTTTATTCTT-3’ |
| *Htr6-F* | 5’-TTCTTCCTGGTGTCGCTCTT-3’ |
| *Htr6-R* | 5’-GGCAGAGGTTGAGAATGGAG-3’ |
| *Hspb1-F* | 5’-CCTCTTCCCTATCCCCTGAG-3’ |
| *Hspb1-R* | 5’-CAAAAGAGCGCACAGATTGA-3’ |
| *Lcn2-F* | 5’-CTGAATGGGTGGTGAGTGTG-3’ |
| *Lcn2-R* | 5’-GCTCTCTGGCAACAGGAAAG-3’ |
| *Caps1-F* | 5’-TCACTCTTGGAACGGGTTTT-3’ |
| *Caps1-R* | 5’-GGCCTACATTTTCTGCATCC-3’ |
| chr2-24463149-F | 5’-GACCCTCTGTGTACGGCAAT-3’ |
| chr2-24463149-R | 5’-TTTATACCGGAGAGGGCAGA-3’ |
| chr7-148629063-F | 5’-GCCTGGAGCACCTTTTCTC-3’ |
| chr7-148629063-R | 5’-GAACCTTGGCTAGGGTGTCA-3’ |
| chr17-46595676-F | 5’-CCTCCCTGCTTTCAGCACT-3’ |
| chr17-46595676-R | 5’-GGTGGTGGAACTCTCCAGAA-3’ |

**Tabel S2. The DEGs of acute morphine model**

| **gene_id** | **sample_1** | **sample_2** | **fpkm_1** | **fpkm_2** | **log2_fold** | **p_value** | **q_value** |
| --- | --- | --- | --- | --- | --- | --- | --- |
| *1200009I06Rik* | Saline | Morphine | 0.60 | 0.15 | -2.01 | 3.79E-05 | 3.54E-03 |
| *3830431G21Rik* | Saline | Morphine | 2.12 | 0.80 | -1.40 | 2.28E-06 | 3.91E-04 |
| *6430704M03Rik* | Saline | Morphine | 12.28 | 24.71 | 1.01 | 6.65E-08 | 1.87E-05 |
| *9430031J16Rik* | Saline | Morphine | 0.52 | 1.06 | 1.04 | 6.41E-04 | 2.68E-02 |
| *A230001M10Rik* | Saline | Morphine | 1.05 | 0.27 | -1.95 | 5.42E-05 | 4.81E-03 |
| *Abi3bp* | Saline | Morphine | 1.45 | 4.35 | 1.58 | 2.53E-09 | 1.19E-06 |
| *Adamts19* | Saline | Morphine | 0.50 | 0.03 | -4.27 | 1.47E-06 | 2.73E-04 |
| *Adcyap1* | Saline | Morphine | 5.94 | 13.33 | 1.17 | 2.13E-07 | 5.37E-05 |
| *Aff2* | Saline | Morphine | 0.51 | 1.08 | 1.10 | 2.46E-04 | 1.40E-02 |
| *Agt* | Saline | Morphine | 21.07 | 9.92 | -1.09 | 2.79E-08 | 8.40E-06 |
| *AI848285* | Saline | Morphine | 5.21 | 1.30 | -2.00 | 1.75E-10 | 1.03E-07 |
| *Akr1b3* | Saline | Morphine | 4.23 | 10.96 | 1.37 | 1.68E-04 | 1.06E-02 |
| *Alx4* | Saline | Morphine | 0.49 | 0.20 | -1.30 | 5.39E-04 | 2.39E-02 |
| *Apoc1* | Saline | Morphine | 5.57 | 1.70 | -1.71 | 6.07E-04 | 2.58E-02 |
| *Atp2b4* | Saline | Morphine | 6.90 | 14.68 | 1.09 | 6.91E-07 | 1.52E-04 |
| *Avp* | Saline | Morphine | 2.05 | 6.35 | 1.63 | 5.61E-04 | 2.47E-02 |
| *Bche* | Saline | Morphine | 1.14 | 0.54 | -1.08 | 3.61E-04 | 1.86E-02 |
| *Bmpr2* | Saline | Morphine | 2.82 | 5.91 | 1.07 | 1.18E-06 | 2.24E-04 |
| *C1ql2* | Saline | Morphine | 3.80 | 1.31 | -1.54 | 6.34E-07 | 1.46E-04 |
| *Cacng5* | Saline | Morphine | 5.41 | 2.58 | -1.07 | 2.36E-05 | 2.48E-03 |
| *Calb2* | Saline | Morphine | 53.13 | 23.94 | -1.15 | 9.01E-10 | 4.54E-07 |
| *Capn6* | Saline | Morphine | 1.01 | 0.35 | -1.53 | 2.33E-05 | 2.47E-03 |
| *Cartpt* | Saline | Morphine | 5.64 | 11.85 | 1.07 | 1.29E-03 | 4.38E-02 |
| *Ccl9* | Saline | Morphine | 0.66 | 1.53 | 1.21 | 6.93E-04 | 2.85E-02 |
| *Cdh6* | Saline | Morphine | 0.84 | 1.76 | 1.07 | 5.96E-04 | 2.57E-02 |
| *Cdhr1* | Saline | Morphine | 6.37 | 1.46 | -2.12 | 0.00E+00 | 0.00E+00 |
| *Cdkl5* | Saline | Morphine | 1.67 | 4.35 | 1.39 | 1.11E-08 | 4.35E-06 |
| *CK137956* | Saline | Morphine | 0.65 | 1.56 | 1.26 | 2.15E-04 | 1.26E-02 |
| *Clca1* | Saline | Morphine | 1.06 | 0.35 | -1.57 | 1.83E-05 | 2.04E-03 |
| *Cmtm4* | Saline | Morphine | 2.12 | 5.69 | 1.43 | 1.00E-05 | 1.34E-03 |
| *Cpa6* | Saline | Morphine | 1.11 | 0.27 | -2.05 | 2.06E-05 | 2.23E-03 |
| *Crlf1* | Saline | Morphine | 1.80 | 0.42 | -2.09 | 4.01E-07 | 9.75E-05 |
| *Cwc22* | Saline | Morphine | 45.59 | 20.56 | -1.15 | 2.72E-08 | 8.40E-06 |
| *Cyp27a1* | Saline | Morphine | 2.66 | 1.14 | -1.23 | 1.72E-04 | 1.08E-02 |
| *Cyp2a5* | Saline | Morphine | 0.75 | 0.09 | -3.13 | 7.44E-05 | 5.93E-03 |
| *Cyp2j12* | Saline | Morphine | 1.54 | 0.61 | -1.35 | 2.72E-04 | 1.50E-02 |
| *Dct* | Saline | Morphine | 0.22 | 0.73 | 1.72 | 4.42E-04 | 2.14E-02 |
| *Dlk1* | Saline | Morphine | 2.11 | 4.44 | 1.07 | 4.77E-06 | 7.31E-04 |
| *Dmrt2* | Saline | Morphine | 0.23 | 0.95 | 2.02 | 2.58E-05 | 2.69E-03 |
| *Doc2g* | Saline | Morphine | 24.31 | 3.88 | -2.65 | 0.00E+00 | 0.00E+00 |
| *Dok6* | Saline | Morphine | 1.96 | 4.84 | 1.30 | 1.42E-04 | 9.21E-03 |
| *Dsp* | Saline | Morphine | 1.55 | 0.08 | -4.25 | 0.00E+00 | 0.00E+00 |
| *Eomes* | Saline | Morphine | 2.90 | 0.44 | -2.74 | 2.22E-16 | 3.13E-13 |
| *Epn3* | Saline | Morphine | 0.73 | 0.14 | -2.42 | 1.30E-08 | 4.81E-06 |
| *Fam117a* | Saline | Morphine | 1.65 | 0.74 | -1.15 | 4.84E-04 | 2.22E-02 |
| *Fam198b* | Saline | Morphine | 0.83 | 1.92 | 1.21 | 7.19E-06 | 1.03E-03 |
| *Fam19a4* | Saline | Morphine | 0.64 | 0.14 | -2.19 | 1.12E-03 | 3.96E-02 |
| *Foxd3* | Saline | Morphine | 0.55 | 0.05 | -3.41 | 1.67E-04 | 1.06E-02 |
| *Frmd7* | Saline | Morphine | 4.14 | 0.73 | -2.50 | 0.00E+00 | 0.00E+00 |
| *Frmpd3* | Saline | Morphine | 0.09 | 0.38 | 2.04 | 9.43E-04 | 3.59E-02 |
| *Fzd4* | Saline | Morphine | 0.82 | 1.67 | 1.03 | 1.01E-03 | 3.73E-02 |
| *Gbp4* | Saline | Morphine | 5.71 | 1.98 | -1.53 | 9.49E-12 | 6.69E-09 |
| *Gbx2* | Saline | Morphine | 0.94 | 0.31 | -1.58 | 2.54E-04 | 1.43E-02 |
| *Gdpd4* | Saline | Morphine | 0.40 | 0.06 | -2.81 | 3.97E-04 | 1.99E-02 |
| *Gfap* | Saline | Morphine | 108.04 | 47.73 | -1.18 | 9.13E-07 | 1.89E-04 |
| *Glra3* | Saline | Morphine | 0.42 | 1.28 | 1.59 | 6.36E-04 | 2.68E-02 |
| *Gm13691* | Saline | Morphine | 6.80 | 2.80 | -1.28 | 1.37E-08 | 4.97E-06 |
| *Gm13693* | Saline | Morphine | 6.85 | 2.82 | -1.28 | 1.26E-08 | 4.80E-06 |
| *Gm13695* | Saline | Morphine | 6.34 | 2.63 | -1.27 | 2.86E-08 | 8.40E-06 |
| *Gm13697* | Saline | Morphine | 6.34 | 2.63 | -1.27 | 2.86E-08 | 8.40E-06 |
| *Gm13698* | Saline | Morphine | 6.34 | 2.63 | -1.27 | 2.86E-08 | 8.40E-06 |
| *Gnrh1* | Saline | Morphine | 0.50 | 2.82 | 2.51 | 3.33E-04 | 1.74E-02 |
| *Gpr26* | Saline | Morphine | 3.06 | 6.38 | 1.06 | 2.59E-05 | 2.69E-03 |
| *Grin3a* | Saline | Morphine | 1.05 | 2.54 | 1.28 | 8.01E-06 | 1.14E-03 |
| *Gucy1a2* | Saline | Morphine | 0.57 | 1.32 | 1.20 | 3.84E-05 | 3.56E-03 |
| *Gucy2g* | Saline | Morphine | 0.75 | 1.86 | 1.30 | 1.51E-05 | 1.79E-03 |
| *Hapln2* | Saline | Morphine | 8.61 | 4.27 | -1.01 | 1.48E-05 | 1.77E-03 |
| *Hba-a2* | Saline | Morphine | 37.57 | 164.44 | 2.13 | 4.38E-10 | 2.47E-07 |
| *Hcrt* | Saline | Morphine | 0.55 | 40.02 | 6.19 | 0.00E+00 | 0.00E+00 |
| *Hgf* | Saline | Morphine | 0.36 | 1.07 | 1.55 | 1.04E-04 | 7.33E-03 |
| *Hkdc1* | Saline | Morphine | 1.73 | 3.78 | 1.13 | 9.47E-06 | 1.31E-03 |
| *Htr2a* | Saline | Morphine | 1.68 | 6.04 | 1.85 | 8.34E-13 | 6.54E-10 |
| *Igsf9b* | Saline | Morphine | 0.65 | 1.41 | 1.12 | 1.65E-03 | 4.94E-02 |
| *Il12a* | Saline | Morphine | 0.45 | 1.33 | 1.55 | 1.29E-03 | 4.38E-02 |
| *Il1f9* | Saline | Morphine | 3.98 | 1.54 | -1.37 | 1.59E-05 | 1.87E-03 |
| *Inadl* | Saline | Morphine | 3.71 | 1.77 | -1.07 | 7.35E-05 | 5.89E-03 |
| *Inhba* | Saline | Morphine | 0.65 | 1.64 | 1.32 | 9.39E-04 | 3.59E-02 |
| *Kcna3* | Saline | Morphine | 0.60 | 1.48 | 1.30 | 8.58E-04 | 3.35E-02 |
| *Kcnh5* | Saline | Morphine | 0.81 | 1.80 | 1.15 | 8.97E-05 | 6.66E-03 |
| *Kcnh7* | Saline | Morphine | 0.84 | 2.51 | 1.58 | 1.92E-08 | 6.61E-06 |
| *Klhl14* | Saline | Morphine | 0.18 | 0.85 | 2.20 | 2.07E-08 | 6.96E-06 |
| *Lct* | Saline | Morphine | 1.02 | 0.20 | -2.36 | 4.14E-12 | 3.07E-09 |
| *Lgals4* | Saline | Morphine | 1.30 | 0.40 | -1.69 | 4.79E-04 | 2.22E-02 |
| *Lgr5* | Saline | Morphine | 2.11 | 0.64 | -1.73 | 6.10E-09 | 2.46E-06 |
| *Lgr6* | Saline | Morphine | 1.11 | 0.32 | -1.80 | 9.49E-07 | 1.91E-04 |
| *Lhx9* | Saline | Morphine | 0.92 | 0.11 | -3.13 | 9.97E-06 | 1.34E-03 |
| *Lipg* | Saline | Morphine | 0.43 | 1.05 | 1.29 | 1.78E-04 | 1.10E-02 |
| *Lmbrd2* | Saline | Morphine | 2.10 | 5.11 | 1.28 | 7.32E-07 | 1.59E-04 |
| *Lrrc17* | Saline | Morphine | 11.07 | 26.78 | 1.27 | 3.05E-11 | 1.87E-08 |
| *Mei4* | Saline | Morphine | 0.10 | 0.39 | 1.98 | 1.42E-03 | 4.62E-02 |
| *Meis1* | Saline | Morphine | 2.41 | 0.85 | -1.50 | 1.80E-06 | 3.17E-04 |
| *Mgat5* | Saline | Morphine | 2.37 | 5.36 | 1.17 | 9.10E-07 | 1.89E-04 |
| *Ms4a15* | Saline | Morphine | 5.95 | 0.58 | -3.35 | 0.00E+00 | 0.00E+00 |
| *Myoc* | Saline | Morphine | 2.94 | 0.81 | -1.85 | 5.35E-09 | 2.22E-06 |
| *Ndst4* | Saline | Morphine | 0.38 | 1.41 | 1.90 | 6.78E-08 | 1.87E-05 |
| *Nmb* | Saline | Morphine | 6.03 | 1.67 | -1.86 | 3.24E-07 | 8.01E-05 |
| *Nmbr* | Saline | Morphine | 0.56 | 2.30 | 2.05 | 1.22E-05 | 1.54E-03 |
| *Nov* | Saline | Morphine | 18.05 | 37.20 | 1.04 | 2.65E-08 | 8.40E-06 |
| *Npr1* | Saline | Morphine | 1.45 | 0.35 | -2.04 | 2.83E-09 | 1.29E-06 |
| *Ntng1* | Saline | Morphine | 5.26 | 2.58 | -1.03 | 4.47E-06 | 7.16E-04 |
| *Ntrk1* | Saline | Morphine | 0.08 | 0.42 | 2.45 | 1.52E-03 | 4.78E-02 |
| *Omp* | Saline | Morphine | 6.60 | 0.98 | -2.75 | 6.57E-04 | 2.72E-02 |
| *Opalin* | Saline | Morphine | 31.72 | 15.80 | -1.01 | 1.76E-06 | 3.14E-04 |
| *Otx2* | Saline | Morphine | 1.05 | 0.13 | -3.01 | 4.56E-06 | 7.23E-04 |
| *Ovgp1* | Saline | Morphine | 2.26 | 0.99 | -1.19 | 1.93E-04 | 1.18E-02 |
| *Padi2* | Saline | Morphine | 6.18 | 3.05 | -1.02 | 6.64E-07 | 1.49E-04 |
| *Pbx3* | Saline | Morphine | 7.51 | 3.52 | -1.09 | 2.14E-06 | 3.73E-04 |
| *Pcdh11x* | Saline | Morphine | 0.43 | 1.25 | 1.54 | 1.70E-06 | 3.08E-04 |
| *Pcsk1* | Saline | Morphine | 2.29 | 5.80 | 1.34 | 1.81E-07 | 4.81E-05 |
| *Pipox* | Saline | Morphine | 1.00 | 0.38 | -1.38 | 1.46E-03 | 4.67E-02 |
| *Pitx2* | Saline | Morphine | 0.16 | 0.84 | 2.41 | 1.05E-03 | 3.82E-02 |
| *Plcxd3* | Saline | Morphine | 0.43 | 1.20 | 1.50 | 3.75E-04 | 1.90E-02 |
| *Plekhg1* | Saline | Morphine | 4.42 | 1.92 | -1.20 | 1.59E-08 | 5.61E-06 |
| *Pmaip1* | Saline | Morphine | 1.03 | 2.31 | 1.16 | 4.48E-04 | 2.15E-02 |
| *Pmch* | Saline | Morphine | 0.74 | 42.55 | 5.85 | 0.00E+00 | 0.00E+00 |
| *Postn* | Saline | Morphine | 0.59 | 1.41 | 1.26 | 1.11E-03 | 3.96E-02 |
| *Ppm1j* | Saline | Morphine | 1.04 | 0.34 | -1.61 | 6.02E-04 | 2.57E-02 |
| *Prkcd* | Saline | Morphine | 11.97 | 3.72 | -1.69 | 7.11E-15 | 7.16E-12 |
| *Prox1* | Saline | Morphine | 4.02 | 1.28 | -1.65 | 1.64E-11 | 1.10E-08 |
| *Prr16* | Saline | Morphine | 1.71 | 3.55 | 1.06 | 1.57E-04 | 1.01E-02 |
| *Rab37* | Saline | Morphine | 4.45 | 0.78 | -2.52 | 4.60E-13 | 3.82E-10 |
| *Rgs4* | Saline | Morphine | 81.62 | 168.53 | 1.05 | 3.01E-05 | 3.00E-03 |
| *Rreb1* | Saline | Morphine | 6.47 | 3.06 | -1.08 | 4.93E-07 | 1.18E-04 |
| *S100a5* | Saline | Morphine | 43.25 | 3.08 | -3.81 | 0.00E+00 | 0.00E+00 |
| *Scgn* | Saline | Morphine | 4.64 | 0.40 | -3.54 | 5.26E-14 | 4.95E-11 |
| *Scnn1a* | Saline | Morphine | 0.74 | 0.26 | -1.50 | 6.91E-05 | 5.77E-03 |
| *Serpinb1a* | Saline | Morphine | 5.49 | 2.12 | -1.37 | 9.43E-07 | 1.91E-04 |
| *Shc3* | Saline | Morphine | 5.33 | 10.67 | 1.00 | 1.01E-05 | 1.34E-03 |
| *Shisa3* | Saline | Morphine | 1.22 | 0.28 | -2.13 | 4.95E-06 | 7.49E-04 |
| *Shox2* | Saline | Morphine | 1.15 | 0.17 | -2.75 | 3.69E-05 | 3.52E-03 |
| *Six3* | Saline | Morphine | 3.66 | 1.64 | -1.16 | 3.77E-06 | 6.29E-04 |
| *Slc26a10* | Saline | Morphine | 1.27 | 0.41 | -1.63 | 1.35E-04 | 8.87E-03 |
| *Slc2a10* | Saline | Morphine | 0.49 | 0.15 | -1.72 | 1.03E-03 | 3.77E-02 |
| *Slc43a3* | Saline | Morphine | 0.58 | 0.17 | -1.78 | 6.82E-04 | 2.81E-02 |
| *Slitrk4* | Saline | Morphine | 1.78 | 3.71 | 1.06 | 2.23E-05 | 2.38E-03 |
| *Smtnl2* | Saline | Morphine | 1.09 | 0.45 | -1.26 | 9.85E-04 | 3.67E-02 |
| *Sox2ot* | Saline | Morphine | 10.15 | 4.93 | -1.04 | 1.89E-04 | 1.16E-02 |
| *Sp7* | Saline | Morphine | 1.72 | 0.31 | -2.48 | 4.73E-10 | 2.57E-07 |
| *Sp8* | Saline | Morphine | 2.15 | 0.93 | -1.21 | 4.63E-05 | 4.24E-03 |
| *Sp9* | Saline | Morphine | 4.21 | 1.91 | -1.14 | 3.79E-06 | 6.29E-04 |
| *Spdef* | Saline | Morphine | 2.97 | 0.09 | -5.10 | 1.27E-13 | 1.12E-10 |
| *Spp1* | Saline | Morphine | 5.34 | 2.00 | -1.42 | 5.13E-06 | 7.62E-04 |
| *St18* | Saline | Morphine | 1.75 | 0.87 | -1.02 | 7.58E-05 | 5.97E-03 |
| *St6gal2* | Saline | Morphine | 1.70 | 3.89 | 1.19 | 3.02E-05 | 3.00E-03 |
| *Stoml3* | Saline | Morphine | 1.43 | 0.33 | -2.13 | 1.69E-03 | 4.96E-02 |
| *Sult1a1* | Saline | Morphine | 3.74 | 7.87 | 1.07 | 6.06E-05 | 5.21E-03 |
| *Tac1* | Saline | Morphine | 11.79 | 23.98 | 1.02 | 1.01E-05 | 1.34E-03 |
| *Tal1* | Saline | Morphine | 0.65 | 0.25 | -1.40 | 3.68E-04 | 1.87E-02 |
| *Tbx21* | Saline | Morphine | 1.28 | 0.06 | -4.31 | 5.94E-10 | 3.10E-07 |
| *Tcap* | Saline | Morphine | 3.01 | 6.48 | 1.11 | 5.05E-04 | 2.28E-02 |
| *Tcf7l2* | Saline | Morphine | 7.51 | 2.47 | -1.60 | 2.58E-11 | 1.65E-08 |
| *Tcfap2d* | Saline | Morphine | 0.06 | 0.61 | 3.23 | 2.03E-04 | 1.23E-02 |
| *Th* | Saline | Morphine | 11.43 | 5.27 | -1.12 | 8.88E-07 | 1.89E-04 |
| *Tmem149* | Saline | Morphine | 3.21 | 1.41 | -1.19 | 9.56E-04 | 3.61E-02 |
| *Tmem196* | Saline | Morphine | 1.99 | 4.35 | 1.13 | 9.45E-04 | 3.59E-02 |
| *Tmem200a* | Saline | Morphine | 1.73 | 3.65 | 1.08 | 1.92E-05 | 2.10E-03 |
| *Tnnc1* | Saline | Morphine | 23.94 | 57.67 | 1.27 | 3.80E-09 | 1.68E-06 |
| *Tnnt1* | Saline | Morphine | 19.55 | 4.30 | -2.19 | 6.66E-16 | 7.83E-13 |
| *Trh* | Saline | Morphine | 7.69 | 2.59 | -1.57 | 5.34E-09 | 2.22E-06 |
| *Trim59* | Saline | Morphine | 4.19 | 2.09 | -1.00 | 2.45E-04 | 1.40E-02 |
| *Tshz1* | Saline | Morphine | 7.68 | 3.46 | -1.15 | 2.22E-09 | 1.08E-06 |
| *Tspan18* | Saline | Morphine | 1.93 | 0.70 | -1.46 | 3.24E-08 | 9.32E-06 |
| *Ttr* | Saline | Morphine | 36.89 | 11.34 | -1.70 | 1.11E-15 | 1.20E-12 |
| *Ush1g* | Saline | Morphine | 0.75 | 0.17 | -2.17 | 1.01E-04 | 7.24E-03 |
| *Vipr2* | Saline | Morphine | 1.86 | 0.25 | -2.87 | 4.44E-16 | 5.69E-13 |

**Table S3. The DEGs of CPP model**

| **gene_id** | **sample_1** | **sample_2** | **fpkm_1** | **fpkm_2** | **log2_fold** | **test_stat** | **p_value** | **q_value** |
| --- | --- | --- | --- | --- | --- | --- | --- | --- |
| *2610305D13Rik* | Saline | Morphine | 0.16 | 0.54 | -1.74 | 3.69 | 2.20E-04 | 1.88E-02 |
| *Btg3* | Saline | Morphine | 11.67 | 5.82 | 1.00 | -6.82 | 9.01E-12 | 6.26E-09 |
| *Chrna3* | Saline | Morphine | 0.17 | 0.57 | -1.77 | 4.50 | 6.95E-06 | 1.44E-03 |
| *Chrnb4* | Saline | Morphine | 0.12 | 0.59 | -2.33 | 6.31 | 2.82E-10 | 1.61E-07 |
| *Col4a3* | Saline | Morphine | 0.17 | 0.39 | -1.15 | 4.93 | 8.22E-07 | 2.50E-04 |
| *Cpt1b* | Saline | Morphine | 0.15 | 0.49 | -1.73 | 3.34 | 8.29E-04 | 4.61E-02 |
| *Elmo3* | Saline | Morphine | 0.29 | 0.64 | -1.14 | 3.73 | 1.93E-04 | 1.73E-02 |
| *Fap* | Saline | Morphine | 0.42 | 1.14 | -1.42 | 4.96 | 7.11E-07 | 2.21E-04 |
| *Gkn3* | Saline | Morphine | 2.11 | 0.71 | 1.57 | -4.06 | 4.99E-05 | 6.32E-03 |
| *Gpr151* | Saline | Morphine | 0.33 | 1.01 | -1.62 | 3.81 | 1.39E-04 | 1.39E-02 |
| *Hspb1* | Saline | Morphine | 4.68 | 2.13 | 1.13 | -5.24 | 1.63E-07 | 6.13E-05 |
| *Htr6* | Saline | Morphine | 1.84 | 0.90 | 1.04 | -3.46 | 5.48E-04 | 3.54E-02 |
| *Lcn2* | Saline | Morphine | 1.10 | 0.19 | 2.55 | -3.61 | 3.11E-04 | 2.40E-02 |
| *Mfrp* | Saline | Morphine | 1.19 | 2.74 | -1.20 | 6.94 | 4.01E-12 | 3.19E-09 |
| *Nhlh2* | Saline | Morphine | 0.38 | 0.77 | -1.02 | 3.71 | 2.09E-04 | 1.82E-02 |
| *Otx2* | Saline | Morphine | 2.30 | 4.87 | -1.08 | 7.11 | 1.16E-12 | 1.03E-09 |
| *Slc16a8* | Saline | Morphine | 0.68 | 1.48 | -1.11 | 3.36 | 7.92E-04 | 4.47E-02 |
| *Slc6a12* | Saline | Morphine | 0.16 | 0.68 | -2.08 | 4.13 | 3.56E-05 | 4.81E-03 |

**Table S4. Summary of second-generation sequencing data in MSCC-seq.**

|  | **Library** | **Number of mapped reads** | **Number of CCGG sites seen at least once** | **Average number of reads per CCGG site** |
| --- | --- | --- | --- | --- |
| CPP model | Morphine-H | 42161728 | 1122229 | 37.6 |
|  | Morphine-M | 20661386 | 1040225 | 19.9 |
|  | Saline-H | 29685409 | 1074481 | 27.6 |
|  | Saline-M | 17785652 | 1052342 | 16.9 |
| Acute model | Morphine-H | 30016958 | 1006928 | 29.8 |
|  | Morphine-M | 26505397 | 1118917 | 23.7 |
|  | Saline-H | 23554197 | 856054 | 27.5 |
|  | Saline-M | 24524888 | 1100297 | 22.3 |
